# Supplementary material for: Review of electrocardiographic abnormalities among people living with HIV in Sub-Saharan Africa: A systematic review
Source: PLoS One. 2023 Mar 23;18(3):e0283419. doi: 10.1371/journal.pone.0283419 (PMC10035850; doi:10.1371/journal.pone.0283419)
Supplement: S1 Appendix — (DOCX) [file pone.0283419.s003.docx]

**S2: SEARCH STRATEGY USED.**

MEDLINE

(("electrocardiogram"[Text Word] OR "cardiac arrhythmias"[Text Word] OR "electrocardio*"[Text Word] OR "12 lead Electrocardiography"[Text Word] OR "EKG"[Text Word] OR "ECG"[Text Word] OR "Long QT Syndrome"[MeSH Terms] OR "arrhythmias, cardiac"[MeSH Terms] OR "Cardiac Conduction System Disease"[MeSH Terms]) AND ("HIV"[Text Word] OR "hiv infection*"[Text Word] OR "HIV"[MeSH Terms] OR "HIV Long-Term Survivors"[MeSH Terms]) AND ("Sub-Saharan Africa"[Text Word] OR "Africa South of the Sahara"[MeSH Terms])) AND (2000:2020[pdat]).

Results: 44

Research 4 Life:

((Subject Terms:("Conduction disorder")) OR (Electrocardiogram) OR (Arrhythmias) OR ("ECG abnormalities")) AND ((Subject Terms:(HIV)) OR ("People living with HIV")) AND ("Sub Saharan Africa")

Results: 125

CINAHL-

"electrocardiogram" OR "cardiac arrhythmias" OR "Cardiac Conduction System Disease" OR ECG OR EKG OR "12 lead Electrocardiography" AND "HIV Long-Term Survivors" OR ( hiv or aids or acquired human immunodeficiency syndrome or human immunodeficiency virus ) AND ( sub saharan africa or sub-saharan africa or sub sahara or sub-sahara )

Results: 46

EMBASE

('cardiac arrhythmias'/exp OR 'cardiac arrhythmias' OR (cardiac AND arrhythmias)) AND ('ekg' OR 'ekg'/exp OR ekg) AND ('hiv' OR 'hiv'/exp OR hiv) AND ('africa south of the sahara'/exp OR 'africa south of the sahara')

Results: 19
