# Supplementary material for: Review of electrocardiographic abnormalities among people living with HIV in Sub-Saharan Africa: A systematic review
Source: PLoS One. 2023 Mar 23;18(3):e0283419. doi: 10.1371/journal.pone.0283419 (PMC10035850; doi:10.1371/journal.pone.0283419)
Supplement: Attachment — Submitted filename: Response to the reviewer.pdf [file pone.0283419.s004.pdf]

MAKERERE UNIVERSITY

MAKNCD RESEARCH TRAINING PROGRAM
Makerere University College of Health Sciences
Makerere University Lung Institute
Email: makncd@chs.mak.ac.ug Tel: +256 700 800 618 / +256 788 391 553

MakNCD Research Training Program

28 December 2021

To the Editor,

PLOS One

Dear Sir/Madam,

RE: <u>Response to comments from the reviewers.</u>

We are thankful to the reviewers and editors for the constructive comments that helped us to considerably improve our manuscript. We have carefully revised the manuscript considering the suggestions and replied to each of the concerns below. We hope that our responses satisfy the reviewers.

Reviewer #1: Overall this is a clear and well-written manuscript synthesizing data about a problem that has been under-studied. Here are my minor comments:

Introduction:

Comments:
"and is highly predictive of incident CVDs (9, 12)"--I do not think this statement is true or justified by the references. Consider rewording--"and can be predictive of incident CVDs"

"Moreover, ECG abnormalities are highly prevalent in the general population and HIV population with the
prevalence of ECG abnormalities ranging from 1.04% to 51% (9, 13-18)." I do not understand this sentence--you seem to be citing several articles about HIV-infected and HIV-uninfected patients in high-income and low-income settings. Overall, I do not think this sentence adds anything and you should consider deleting it.

"With an increased population aging amongst PLWH" Consider re-writing in clearer English

Response:

Thank you for the comment. These have been rectified.

Methods:

Comments
Page 13, line 95: "develop" should be "developed"
Line 151: missing a space between sentences
Line 163: remove the word "the"
Line 170: I don't understand this sentence "three studies, which were the majority"-- three is not the majority of 7.

Response:

Thank you for the comments. These have been rectified.

Comment:
I find it very confusing to combine the pediatric data with the adult data, since you would expect VERY different prevalences of ECG abnormalities among these populations. I would consider breaking this into two separate tables with separate summary statements.

Response:

Thank you for the comment. A separate table for pediatric data has been created.

Discussion:

Comments:
I think the discussion is missing several points and needs to be heavily re-written. Most of the Discussion consists of the authors citing random studies from other settings but not really synthesizing the data the presented in their results in a meaningful way.
-You report a HUGE range of ECG abnormalities (6-81%) which is so large that the data is almost unhelpful. Please comment on why you think this range is so large with specific reference to study design, study population, etc. Since the studies evaluated different sets of ECG abnormalities, I don't think it's helpful to report this summary range because each study reported different kinds of ECG abnormalities.

Response: Thank you for comment. An explanation for the wide range has been added in line 219 to 243.

Comment:
-I think separating out the adult and pediatric studies would be more helpful for your discussion of the adults since the findings and clinical implications are so different.

Response: Thank you for the comment. Different tables have been created. Table 2 and Table 4 to cover the pediatric population.

Comments:
-I suggest you delete this sentence: "In a prospective cohort study involving 1,770 HIV-infected and 3350 HIV negative
participants, ECG abnormalities were found in 35% of the HIV patients which was higher than 32.2% found in HIV negative individuals (43). This difference was statistically significant (p=0.04)." This adds nothing to your discussion, since you already reported results from a similar study in China (where this study was done). You also don't report comparative data between HIV-infected and HIV-uninfected so this is not relevant to your findings.
-When you discuss the SMART study, please specify which countries/regions this was in.
-Overall I think all of Paragraph 2 of the Discussion could be collapsed to a single sentence: "The prevalence of ECG abnormalities among ADULTS in our study was similar to what has been reported in high income settings" -Line 210, remove the word "likely"
-Lines 212 to 219, please specify which country each of these cited studies were in.
-I suggest deleting lines 220-229, as these are redundant from the introduction.
-"ECG are easily accessible to most settings in SSA and could play a huge role in identify PLWH at a risk of developing severe CVDs if used routinely." I think both parts of this sentence are untrue. ECGs are certainly not widely available to people with HIV in a lot of parts of SSA. I don't think ECGs could identify those at risk of developing CVDs--more accurate to say that they could identify some forms of CVD such as arrhythmias and ischemia?
-"Furthermore, it may play an important role in determining the prognosis of PLWH with suspected cardiovascular diseases." This statement is untrue.
-Again, I think a separate paragraph about pediatric results would be helpful.
-Line 234, re-write as "which may have led to exclusion of other studies reporting ECG abnormalities among persons with HIV in SSA"
-Line 235, re-write as "which likely resulted in significant heterogeneity in results"
-Line 238: I don't think it's fair to say this review presents an "exact burden" as the ranges are so large and there is so much heterogeneity among studies.

Response: Thank you for the comments. These have been updated.

Comment:
-Please re-write all three Conclusion sentences as the written English is hard to follow.

Response: Thank you for the comment. The conclusion has been re-written.

Reviewer #2:

This is an important study that highlights a significant topic in which there is a large paucity of data in SSA. The manuscript will contribute to the growing body of cardiovascular disease literature in SSA and highlights the burden of disease and need

for further action.

2. While the study appears to be sound, the language is unclear in some areas, making the manuscript difficult to follow at times. I would advise close review of the manuscript to ensure clarity and accuracy in the writing. I have included few lines below for some suggested changes for clarity.

Comments
Line 65 – would rephrase '78% of PLWH could have developed a CVD' to '78% of PLWH may develop CVD'.

Line 76 – would change 'such as arrhythmias, myocardial…' to 'arrhythmias and myocardial…'

Line 79-81 – would rephrase 'With an increased population aging amongst PLWH, the prevalence of ECG abnormalities…' to 'With an increasingly aging population, the prevalence of ECG abnormalities among PLWH is likely to increase…'.

Line 137 – would change 'Rate' to lower case 'rate'

Line 149 – would rephrase 'The studies were done in 7 African countries…' to 'The studies were conducted in 7 African countries'.

In general, would rephrase the term 'commonest' to 'most common' (lines 158, 167)

Lines 170-171 – would rephrase '3 (43%) studies, which were the majority, reported ST elevation whose prevalence was 1.7%...' to '3 (43%) studies, reported ST elevation with prevalence between 1.7%...'

Lines 206-210 are unclear and require rephrasing.

Line 230 – would change 'huge role in identify PLWH' to 'huge role in identifying PLWH'

Response: Thank you for the comment. These have been updated.

Comment:
3. HIV-related characteristics of samples from the 17 studies are not described including duration of HIV, stage of HIV (i.e. well controlled/virologically suppressed vs advanced/untreated), current CD4, viral load (if available), if on ART, the type/class of ART, and the duration of ART. In addition, HIV-related characteristics were not taken into consideration in either the analysis or in the discussion. As HIV-related

characteristics may be directly correlated with ECG abnormalities this is a critical piece that is missing.

Response: Thank you for the comment. These sections have been added to the tables.

4. The authors do not comment on the very wide range of ECG abnormalities found and highlight only the higher range of prevalence. I would suggest commenting on the wide range in the discussion including thoughts about why such a wide range was found, and also discussing how the wide range impacts accuracy and the interpretation of findings.

Response: Thank you for comment. An explanation for the wide range has been added in line 219 to 243.

5. It is interesting that the authors include ECG abnormalities for both children and adults. As these populations are inherently different and likely have different types of cardiovascular disease, it might be interesting to describe if there were any major differences found between children and adults.

Response: Thank you for the comment. Different tables have been created. Table 2 and Table 4 to cover the pediatric population.

6. The authors conclude that there should be increased efforts at including ECG assessment for PLWH in SSA, however, there is no discussion regarding the multiple structural complexities that may prevent wide-scale implementation of ECG screening. I would suggest including in the discussion the logistic and structural barriers regarding the use of ECGs in SSA. For example, are ECG machines really widely available in all settings? Can patients afford the cost of an ECG if an ECG machine is available? Are there health care workers that are readily available and trained to read and interpret ECGs? If an ECG abnormality is found, are health care providers trained regarding next steps in management and are the resources available for providing those next steps in treatment?

Response: Thank you for the comment. This has been updated.

7. In the tables I suggest changing the term HAART to ART and would also not use the term ‘positives’ and instead rephrase to PLWH or persons with HIV. In addition, would consider use of abbreviations in the table with a legend/foot note. It is also unclear to me how the studies are organized in the tables which makes it somewhat difficult to follow. It might be helpful to organize the studies in a systematic manner and would consider organizing the studies by type of ECG abnormality or perhaps by country/region.

MAKERERE UNIVERSITY
MAKNCD RESEARCH TRAINING PROGRAM
Makerere University College of Health Sciences
Makerere University Lung Institute
Email: makncd@chs.mak.ac.ug Tel: +256 700 800 618 / +256 788 391 553
MakNCD Research Training Program

Response: Thank you for the comment. This has been updated.

8. The studies by Hughes J (2019) and Mayosi BM (2006) appear to describe ECG abnormalities among persons with MDR TB and TB pericarditis. The study by Berhe N (2001) describes ECG findings for patients with visceral leishmaniasis. The ECG abnormalities from these studies may be directly related to the severity of their underlying critical illness (TB/leishmaniasis) and may not be associated with HIV. I would suggest commenting on this in the discussion or including this as a limitation.

Response: Thank you for the comment. This has been included in the limitations line 321- 324.

Yours faithfully,

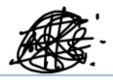

ANDREW WEIL SEMULIMI
