# Supplementary material for: Review of electrocardiographic abnormalities among people living with HIV in Sub-Saharan Africa: A systematic review
Source: PLoS One. 2023 Mar 23;18(3):e0283419. doi: 10.1371/journal.pone.0283419 (PMC10035850; doi:10.1371/journal.pone.0283419)
Supplement: Attachment — Submitted filename: ecg abnormaliteis in PLWH.docx [file pone.0283419.s005.docx]

It was pleasure reviewing the manuscript REVALENCE OF ELECTROCARDIOGRAPHIC ABNORMALITIES AMONG PEOPLE LIVING WITH HIV IN SUB-SAHARAN AFRICA: A SYSTEMATIC REVIEW

**Summary comments**

This is a welcome study that summarizes a growing body of evidence on the unique electrocardiographic characteristics among patients with HIV. The authors responses to prior comments are well noted including highlighting limitations of the study, separation of pediatric studies from adult and description of baseline characteristics of patients enrolled in the studies of interest. The language and phrasing is improved and would recommend the following minor changes.

1. Title: suggest re-framing title to “review of” rather than “prevalence of” electrographic abnormalities among people …..
2. Abstract: concise; would include the total number of retrieved studies and a statement on the limitations of the study in the abstract.
3. Introduction: well focused
4. Methods: appropriate description
5. Results: good descriptions;

- Given the small number of studies, it may be worthwhile reporting in the text, the commonest definition used for each type of abnormality considered eg criteria for LVH, basis for QTc estimations, which ST segment changes were considered ( elevation only, depression, t-wave inverstion etc) between studies.

6. Discussion

- Would reframe as a review of estimates rather than precise prevalence since this was not a meta- analysis at individual patient level bur rather summary descriptions of a collection of studies. For example, would rephrase opening statement as “….our systematic review provides a comprehensive review of published ECG abnormalities among PLWH. In these studies ECG abnormalities ranged between 6.7% -81%.”

7. Limitations

- Appropriate

8. Conclusion

- Concise; Would de-emphasize prevalence and
