# Supplementary material for: Review of electrocardiographic abnormalities among people living with HIV in Sub-Saharan Africa: A systematic review
Source: PLoS One. 2023 Mar 23;18(3):e0283419. doi: 10.1371/journal.pone.0283419 (PMC10035850; doi:10.1371/journal.pone.0283419)
Supplement: Attachment — Submitted filename: Response to the reviewers.docx [file pone.0283419.s006.docx]

To the Academic Editor,

PLOS One

**Response to the reviewers’ comments.**

Reviewer #2: Overall, I think this paper is improved, and the authors are highlighting a very important topic that will significantly contribute to the cardiovascular literature in PLWH in SSA. There are some minor changes needed for clarity in language and writing in the abstract, methods, and results section. I do think the discussion needs to be heavily re-written in many areas and that perhaps there should be more of an emphasis on the clinical significance and implications of the findings.

**Answer: Dear Reviewer, thank you for the comments. The authors have revised the manuscript and changes have been made with more emphasis put on the clinical significance and implications of ECG abnormalities.**

I have included some minor comments for clarity in language and writing below.
Line 43 – I would delete (n-13), this is unnecessary as ‘Thirteen studies’ is stated immediately prior

**Thank you for the comments. The comment has been addressed and the manuscript updated.**
Line 44 – I would delete (n=4) for same reasoning as above

**Thank you for the comments. The comment has been addressed and the manuscript updated**.
Line 56-57 – ECG is not part of ‘risk assessment’ for CVD, but rather a tool to determine existing cardiac abnormalities. Perhaps consider changing this sentence to something like this: ‘Consideration of ECG in the comprehensive evaluation for cardiac dysfunction in PLWH in SSA may be warranted.’

**Thank you for the comments. The comment has been addressed and the manuscript updated.**
Line 101 – Remove the comma after the word Science

**Thank you for the comments. The comment has been addressed and the manuscript updated.**
Line 121 – ‘(Figure 1)’ should be included before the period - i.e. ‘included in the review (Figure 1).’

**Thank you for the comments. The comment has been addressed and the manuscript updated.**
Line 125 – Change ‘that arose were’ to ‘that arose was…’

**Thank you for the comments. The comment has been addressed and the manuscript updated.**
Line 143 – Change ‘ischemia/ Infarction’ to ‘ischemia/infarction’

**Thank you for the comments. The comment has been addressed and the manuscript updated.**
Line 150 – There should be a space between Figure and 1

**Thank you for the comments. The comment has been addressed and the manuscript updated.**
Line 150 – I would remove (n=4)

**Answer: Thank you for the comments. The comment has been addressed and the manuscript updated**.
Lines 159-161 – These sentences are confusing and need clarification. I am uncertain why both the mean and the median age of pediatric participants are reported. Since the mean age of adults is reported, it is reasonable to also report the mean age of pediatric participants, but there is no indication to report the median as well. In addition, I assume (6.3) and (6.6) are the standard error? However, the standard error for pediatric participants is reported differently with a ‘+/-‘. Consistency and clarification are needed in reporting these measures. Also, based on the wording of these sentences, I am uncertain what Table 1 and Table 2 show. I would make this clearer somewhere in this paragraph (i.e. Table 1 describes studies reporting ECG abnormalities in adults)

**Thank you for the comment. The changes have been made, see below:**

“**The mean age of the adult participants ranged between 32±10.5 and 48±13.1 years (Table 1: Studies reporting ECG abnormalities in adult PLWH in SSA) while that of the pediatric participants was between 8.30±3.92 and 8.41±3.99 years (Table 2: Studies reporting ECG abnormalities in pediatric PLWH in SSA).”**

Line 163 – Remove the space after the number 3

**Thank you for the comment. The comment has been addressed and the manuscript updated.**
Line 165 – There is no number for the table

**Thank you for the comments. The sentence has been deleted.**
Lines 170–193 – I am uncertain why the number of studies is repeated after it is written out throughout the results section. For example, in Line 171, ‘Nine (9)’ and in line 174 ‘five (5)’. Was this meant to be the percentage instead of repeating the numbers again?
Lines 195-197 – This sentence is a bit unclear. Consider rewording to something like this: ‘Rate abnormalities were the most commonly reported ECG abnormalities (n=X), followed by atrial and ventricular enlargement (n=x), and lastly conduction abnormalities (n=x)

**Thank you for the comment. This has been adjusted, read below:**

“**followed by atrial and ventricular enlargement (n=3), and lastly conduction abnormalities (n=3)”**
Line 197 – I am uncertain what Table 4 shows. Perhaps consider stating in the beginning of this paragraph, ‘Table 4 describes…’

**Thank you for the comment. The comment has been addressed and the manuscript updated**.
Line 199 – Ventricular should have a lower-case v

**Thank you for the comment. The comment has been addressed and the manuscript updated.**
Lines 201-202 – There should be a comma after prolongation and another comma after 18%

**Thank you for the comment. The comment has been addressed and the manuscript updated.**
Line 208 – There should be a % sign directly after 26.5, and the % after the word children should be deleted.

**Thank you for the comment. The comment has been addressed and the manuscript updated.**

**Discussion**
The discussion needs to be re-written in some areas. While there is now a discussion re: the reason for such a wide range in prevalence of abnormalities, I think that there could be more emphasis in the discussion re: the clinical significance of some of the most common ECG abnormalities. Perhaps focusing less on the overall prevalence and instead focusing more on the clinical significance and real-world implications of some of the major ECG abnormalities in the discussion would be more meaningful.

I’ve included a few specific comments for each of the paragraphs in the discussion below.
I am uncertain what the takeaway message is and am uncertain what the significance of the findings are after reading the first paragraph.

**Thank you for the message: The aim of the paragraph was to highlight the key findings from our review.**

In the second paragraph – I think India is considered an LMIC and I would consider deleting that sentence.

**Thank you for the comment. The comment has been addressed and the manuscript updated.**
The third paragraph is disorganized and difficult to follow. I think the takeaway of the third paragraph is that the wide range in prevalence seen in the ECG abnormalities are due to the significant differences between study samples and discrepancies in ECG interpretation in each study. Perhaps finding a way of stating this more directly and making these points clearer instead of citing and describing the characteristics of each study would be helpful.

**The third paragraph has been edited and reads as below:**

**“The wide range of ECG abnormalities could be attributed to the heterogeneity of the results and the differences in the study population. Regarding the discrepancies in the ECG reporting, several criteria such as American Heart Association Standard criteria (38), normal ECG standard for infants and children (39) and Minnesota Coding system for ECG classification (40) were used by the different studies which may have influenced the prevalence reported. In addition, the characteristics of participants included in the reviewed studies could have contributed to the reported wide range. For instance, Okoye and colleagues (31) studied PLWH who were ART naïve, aged 15 years and above with 30% of their study participants having a low CD4 count (<200 cells/µl) while in a cross-sectional study conducted in Ghana (21), most of the participants had stage III severity of disease with more than half of them (194, 56.3%) having >1 CVD risk. Among the studies conducted in the pediatric population, factors such as wasting (25) and diseases severity (28) influenced results contributing to a wider range in the prevalence of ECG abnormalities. Therefore, the absence of a uniform and standardized tool that can be adjusted for patient characteristics calls for the development of such tools to classify ECG abnormalities. This could inform risk stratification of PLWH at a risk of developing CVDs.”**
Paragraphs 5 and 6 discussing atrial and ventricular enlargement and rate abnormalities are also disorganized and difficult to follow. I might consider focusing on the clinical significance and implications of these findings in these paragraphs.

**The fifth paragraph has been edited and reads as below:**

**“Regarding atrial and ventricular enlargement in adults, findings from our study are similar to those of a cross-sectional study done in China, which reported a prevalence of left ventricular hypertrophy of 6.8% (13). In children, left ventricular hypertrophy was the second most common ECG abnormality. HIV and opportunistic infections coupled with other risk factors of CVDs such as smoking cause inflammation and release of cytokines which have been shown to predispose PLWH to atrial and ventricular hypertrophy (48). This increases the likelihood of individuals to developing ventricular and atrial arrhythmias (49), congestive heart failure and ischemic heart disease (50) which significantly contribute to CVD related mortality and morbidity.”**

**The sixth paragraph has been edited and reads as below:**

**“ECG abnormality among children unlike in the adult population. This was lower than what was reported in the United States of America (USA) (51) which was conducted before the introduction of combined ART. The higher prevalence in the USA study could be attributed to the increased risk of children to opportunistic infections subsequently leading to CVDs (52). HIV instigates viral induced myocarditis which is one of the drivers of sinus tachycardia (53) among PLWH. Sinus tachycardia predisposes patients to life threatening arrhythmias, sudden cardiac death and myocardial infarction and its presence necessitates frequent cardiac monitoring.”**

**“From the reviewed studies, atrial fibrillation is the most reported arrhythmia which is concordant with results from other large epidemiological studies (13, 54, 55). Studies have shown a relationship between HIV and atrial fibrillation although the underlying mechanism has not been fully elucidated (56). Atrial fibrillation causes disruptions in atrial filling and emptying leading to poor clinical outcomes such as cerebrovascular accidents, decompensated congestive cardiac failure, ischemia and sudden cardiac death causing significant morbidity and mortality in this population (29). This highlights how essential ECGs are in diagnosing PLWH with existing cardiac abnormality and dysfunction if used routinely. However, in SSA, the routine use of PLWH to ECGs is marred by the cost and the need to train health workers to read and analyse them (57).”**
Paragraph 7 – I don’t think ECGs are used to identified people who may be at risk for developing CVDs in the future, but instead are used to diagnose existing cardiac abnormality and dysfunction.

**Thank you for the comments. This has been updated.**
Tables
I find some of the tables difficult to read.

I think the titles of the tables need to be revised. For example, instead of the title ‘A table showing the characteristic of the adult review studies’ – perhaps consider changing to ‘studies reporting ECG abnormalities in adult PLWH in SSA’

**Thank you for the comment: The table title has been changed.**
In Table 1 and 2 – I’m uncertain what the p-values included in the column reporting age signifies. I also don’t think the column for HIV diagnosis in Table 1 adds anything and I’d consider deleting this. The column for ECG criteria is largely blank – and for one study the authors state ‘a standard criteria is used’. I’m uncertain what the standard criteria for ECG interpretation is. Are these left blank because the studies did not report how they interpreted the ECG? If so, I would consider deleting this column and reporting this in the results and also including this in the discussion section when discussing reasons for heterogeneity.

**Thank you for the comment. The column on ECG criteria has been removed and the criteria used have been discussed in the discussion.**
In Tables 3 and 4 – I am also uncertain what the p-values included signify.

**Thank you for the comment. The p-values have been deleted.**

Reviewer #3: It was pleasure reviewing the manuscript REVALENCE OF ELECTROCARDIOGRAPHIC ABNORMALITIES AMONG PEOPLE LIVING WITH HIV IN SUB-SAHARAN AFRICA: A SYSTEMATIC REVIEW
Summary comments
This is a welcome study that summarizes a growing body of evidence on the unique electrocardiographic characteristics among patients with HIV. The authors responses to prior comments are well noted including highlighting limitations of the study, separation of pediatric studies from adult and description of baseline characteristics of patients enrolled in the studies of interest. The language and phrasing is improved and would recommend the following minor changes.
1. Title: suggest re-framing title to “review of” rather than “prevalence of” electrographic abnormalities among people …..

**Thank you for the comment. The title of the review has been updated.**
2. Abstract: concise; would include the total number of retrieved studies and a statement on the limitations of the study in the abstract.

**Thank you for the comment. A statement on the limitations of the review has been included in the abstract. “The heterogeneity in results could be attributed to the absence of uniform criteria to define ECG abnormalities.”**
3. Introduction: well focused

4. Methods: appropriate description

5. Results: good descriptions;

- Given the small number of studies, it may be worthwhile reporting in the text, the commonest definition used for each type of abnormality considered eg criteria for LVH, basis for QTc estimations, which ST segment changes were considered (elevation only, depression, t-wave inversion etc) between studies.

**Thank you for the comment. We have included the commonest definitions used to define ECG abnormalities in the reviewed studies. “In the reviewed studies, the most reported definition of ECG abnormalities was QTc interval prolongation defined as more than 0.44 seconds (n=4) (22, 25, 27, 33). Regarding ST changes, ST elevations were the most reported (n=4).”**
6. Discussion
- Would reframe as a review of estimates rather than precise prevalence since this was not a meta- analysis at individual patient level bur rather summary descriptions of a collection of studies. For example, would rephrase opening statement as “….our systematic review provides a comprehensive review of published ECG abnormalities among PLWH. In these studies, ECG abnormalities ranged between 6.7% -81%.”

**Thank you for the comment. The statement been updated.**
7. Limitations
- Appropriate

8. Conclusion
- Concise; Would de-emphasize prevalence and

**Thank you for the comment. The statement been updated.**
