# Supplementary material for: Review of electrocardiographic abnormalities among people living with HIV in Sub-Saharan Africa: A systematic review
Source: PLoS One. 2023 Mar 23;18(3):e0283419. doi: 10.1371/journal.pone.0283419 (PMC10035850; doi:10.1371/journal.pone.0283419)
Supplement: Attachment — Submitted filename: Response to the reviewes.docx [file pone.0283419.s008.docx]

Response to the Editor’s comments

**How could with a similar registration number (CRD42021243664) in research Square (**[**https://doi.org/10.21203/rs.3.rs-743165/v1**](https://doi.org/10.21203/rs.3.rs-743165/v1)**)**

Thank you for the comment. Indeed, an online article titled “Prevalence of Electrocardiographic Abnormalities Among People Living With HIV/AIDS in Sub-Saharan Africa: Protocol for a Systematic Review and Meta-analysis” with the same registration number CRD42021243664 was published in research square. However, kindly note that the said article is a pre-print which did not undergo peer review. Secondly, the article is the protocol for the systematic review and meta-analysis as it is stated clearly in the title. It is a common practise for journals to publish protocols of Systematic reviews and meta-analysis, case in point:

<https://journals.plos.org/plosone/s/submission-guidelines#loc-lab-protocols>

<https://bmjopen.bmj.com/pages/authors>

<https://systematicreviewsjournal.biomedcentral.com/submission-guidelines/preparing-your-manuscript/protocol>

**2-What was the main significance of publishing solely systematic review (PLOS One), and systematic review and Meta analysis (research square/preprint)?**

Thank you for the comments. We submitted and are hoping to publish this paper as a systematic review and not as systematic review and Meta analysis as intended in the protocol because there were no uniform criteria to define ECG abnormalities. This made the pooling of the data retrieved from the published papers difficult making it impossible for us to conduct a meta-analysis which was stated as a limitation, line 272—273.

**Journal Requirements:**

**Please review your reference list to ensure that it is complete and correct. If you have cited papers that have been retracted, please include the rationale for doing so in the manuscript text or remove these references and replace them with relevant current references. Any changes to the reference list should be mentioned in the rebuttal letter that accompanies your revised manuscript. If you need to cite a retracted article, indicate the article’s retracted status in the References list and also include a citation and full reference for the retraction notice.**

Thank you for the comment. The reference list is up to date and no changes have been made to it.
