# Supplementary material for: Review of electrocardiographic abnormalities among people living with HIV in Sub-Saharan Africa: A systematic review
Source: PLoS One. 2023 Mar 23;18(3):e0283419. doi: 10.1371/journal.pone.0283419 (PMC10035850; doi:10.1371/journal.pone.0283419)
Supplement: Attachment — Submitted filename: Decision letter.docx [file pone.0283419.s007.docx]

Journal: PLOS ONE

Title: **Time to death and predictors of mortality among preterm newborns admitted at hospitals in Guraghe Zone, Southern Ethiopia; prospective cohort study**

Number/Id: **PONE-D-21-27823R2**

**Decision: Reject**

I have assessed your manuscript and regret to inform you that it cannot be considered for publication as it does not meet the criteria for PLOS ONE. If I discovered the following core issues, as did both reviewers, we conclude that these findings were not met and, as a result, we are unable to consider your manuscript further. Even though the study appears to be retrospective, it was conducted as a prospective cohort study, which has feasibility issues. Major errors in grammar and logical flow, for example, what were appropriate measures to ensure confidentiality while securing data? In addition, sampling and sampling techniques weren’t well reported. More than 1,000 babies (including preterm babies) will be admitted to the NICU from 6,000 deliveries. So, how could it be that 552*4= more than 2000 preterm annual? Iron deficiency anemia is an example of a variable that was listed in the study variables but was not reported in the result section.
